# Carbon sequestration costs and spatial spillover effects in China's collective forests

**DOI:** 10.1186/s13021-024-00261-5

**Published:** 2024-04-26

**Authors:** Yifan Zhou, Caixia Xue, Shuohua Liu, Jinrong Zhang

**Affiliations:** College of Economics and Management, Northwest A&F University, Xian, 710000 China

**Keywords:** Carbon sequestration costs, Deep neural network, Collective forests, Spatial spillover, Carbon neutrality

## Abstract

**Background:**

Global climate change is one of the major challenges facing the world today, and forests play a crucial role as significant carbon sinks and providers of ecosystem services in mitigating climate change and protecting the environment. China, as one of the largest developing countries globally, owns 60% of its forest resources collectively. Evaluating the carbon sequestration cost of collective forests not only helps assess the contribution of China’s forest resources to global climate change mitigation but also provides important evidence for formulating relevant policies and measures.

**Results:**

Over the past 30 years, the carbon sequestration cost of collective forests in China has shown an overall upward trend. Except for coastal provinces, southern collective forest areas, as well as some southwestern and northeastern regions, have the advantage of lower carbon sequestration costs. Furthermore, LSTM network predictions indicate that the carbon sequestration cost of collective forests in China will continue to rise. By 2030, the average carbon sequestration cost of collective forests is projected to reach 125 CNY per ton(= 16.06 Euros/t). Additionally, there is spatial correlation in the carbon sequestration cost of collective forests. Timber production, labor costs, and labor prices have negative spatial spillover effects on carbon sequestration costs, while land opportunity costs, forest accumulation, and rural resident consumption have positive spatial spillover effects.

**Conclusion:**

The results of this study indicate regional disparities in the spatial distribution of carbon sequestration costs of collective forests, with an undeniable upward trend in future cost growth. It is essential to focus on areas with lower carbon sequestration costs and formulate targeted carbon sink economic policies and management measures to maximize the carbon sequestration potential of collective forests and promote the sustainable development of forestry.

## Introduction

Human activities have caused significant greenhouse gas emissions, leading to the global challenge of climate change [1–3].The negative externalities and spillover effects of these emissions necessitate international cooperation among governments to address climate change [4, 5]. In September 2020, China introduced the “Dual Carbon” goals: peaking carbon emissions by 2030 and achieving carbon neutrality by 2060. This involves two mechanisms: emission reduction and carbon sequestration [6, 7]. Industrial emission reduction technologies can mitigate around 80% of carbon emissions, but the remaining 15–20% reduction poses significant cost challenges [8, 9]. In contrast, forest carbon sequestration within the carbon sequestration mechanism offers cost-effective solutions and positive externalities, such as water conservation, air purification, and environmental improvement [10–12]. Therefore, promoting forest carbon sinks and harnessing their potential presents an economically viable solution to offset the remaining 15–20% of carbon emissions [13, 14].

In China, collective forests refer to forest resources collectively owned and managed by farmers, who possess rights to their use, management, and benefits. These forests play a crucial role in China's ecosystem and hold significant socioeconomic importance for rural livelihoods and development [15]. Therefore, in this paper, ‘‘collective forests’’ denote forest resources owned and managed collectively by farmers [16]. Based on the data from the Ninth National Forest Resource Inventory, the collective forests in China cover an area of 133.9 million hectares, accounting for 61.3% of the total land area. However, the forest stock in these collective forests is 6935.2 billion cubic meters, representing only 40.7% of the total. One of the reasons for the relatively high proportion of collective forest area but low forest stock is the lack of sound management strategies, resulting in poor forest quality and low carbon density in collective forests. Data indicates that the forest stock per hectare in collective forests is merely 51.8 cubic meters, compared to 119.9 cubic meters in state-owned forests, highlighting a significant disparity. In terms of forest age, young and middle-aged forests account for 44.3% and 31.8% respectively in collective timber forests, indicating their significant potential for carbon sequestration.The new round of collective forest tenure reform implemented since 2003 has clarified property rights and activated the management vitality of farmers in collective forests [16]. This provides an institutional foundation for realizing their carbon sequestration potential. Compared to state-owned forests, collective forests have more flexible management forms and unique advantages in carbon sequestration, with higher marginal sequestration and greater overall potential. However, as collective forests are managed by farmers who act as rational economic agents, the cost of carbon sequestration significantly influences their sequestration potential. Furthermore, spatial spillover effects are also important factors influencing carbon sequestration in collective forests. For example, the implementation of effective carbon sequestration policies in certain areas may generate positive spatial spillover effects, prompting neighboring regions to take corresponding measures, thereby further enhancing the carbon sequestration capacity of the entire area.

This paper examines the carbon sequestration potential on China's collective forests, in particularl addressing the economic potential of carbon sequestration costs in China’s collective forests. With China spanning tropical and sub-frigid zones, there are significant natural variation in land quality and growth potential. We thus also consider how this regional variation affects the costs of developing carbon sequestration resources. This analysis allows us to determine which regions have advantages for concentrated development of forest carbon sequestration. By addressing these factors and leveraging the innovative approach of applying deep neural network models to forecast carbon sequestration costs, we are able to consider the future prospects and long-term cost trends for carbon sequestration in collective forests. Finally, we examine spatial correlation and spillover effects in China’s forest carbon sequestration resources. In particular, gaining insight into potential spatial spillover effects in the carbon sequestration costs of collective forests will provide practical help for policy makers developing carbon sequestration resources, improving the effectiveness of forest carbon management, and achieving the ‘‘Dual Carbon’’ goals in the forestry industry in China.

## Literature

Forest carbon sequestration costs encompass the economic losses or opportunity costs incurred to achieve forest carbon sequestration goals [17–19]. Typically measured in monetary units per ton of carbon dioxide, these costs vary due to differences in calculation methods, geographical scope, and underlying assumptions [20]. Existing literature on forest carbon sequestration costs can be categorized into two main perspectives.The first approach adopts an engineering cost perspective, utilizing income and production cost data from representative land types or locations to determine the costs of forest carbon sequestration. This includes considering profit losses from converting land from agricultural to forestry use and the dynamic income from the timber market [21–23]. The second method estimates carbon sequestration costs based on evidence of landowners’ behavior when confronted with the opportunity costs of alternative land uses [24]. Despite differences, both methods share common components, such as the opportunity cost of land conversion and the present value of future revenue from forestry products.Moreover, studies have explored strategies to minimize the costs of forest carbon sequestration, offering practical insights into optimizing carbon sequestration in forest ecosystems [25, 26]. These methodologies have been widely applied, providing valuable recommendations for future forest carbon sequestration projects [27–31]. They have also been instrumental in comparing the costs of forest carbon sequestration with other industrial emission reduction and carbon storage technologies [32, 33]. For instance, scholars have evaluated the economic feasibility of Clean Development Mechanism (CDM) carbon offset projects under different rotation conditions, employing an engineering perspective [34].

Forest carbon sequestration costs are influenced by forest resource endowment, technological progress, and economic development. Research on the factors affecting forest carbon sequestration costs is one of the key themes in current studies [35]. Forestry management technology is a critical anthropogenic factor in forest carbon sequestration [36, 37]. Improved management technology can enhance forestry operational efficiency and reduce operational costs. Conversely, poorly managed forests with imbalanced stand structures, excessive foliage, and accumulation of forest litter and deadwood can lead to increased forest carbon emissions, accompanied by increased management costs [38, 39]. The level of input costs for land, labor, materials, and other resources in forest carbon sequestration activities is closely associated with the level of socioeconomic development. Regions with higher economic development often incur higher costs for forestry operations, labor, and seedling nurturing, resulting in relatively higher total forest carbon sequestration expenses compared to regions with lower economic development levels [40]. The abundance of forest resources to some extent determines the feasibility of carbon sink resource development. Regions endowed with abundant forest resources are often naturally advantaged areas, where superior natural conditions contribute to higher productivity and lower forest carbon sequestration costs. Moreover, the spatial concentration of forest resources leads to spatial spillover effects among various factors [41–43].

Compared to existing literature, this article makes three contributions. First, regarding ownership, Chinese forests are classified into state-owned forests and collectively-owned forests, which may have different carbon sequestration costs due to variations in management entities. Existing studies estimate the carbon sequestration costs as a “mixture” of both types, whereas this article focuses on calculating the carbon sequestration costs specifically for collectively-owned forests. It evaluates the costs by considering afforestation and opportunity costs over the past 30 years.

Second, based on the calculations presented in this paper, we further utilize a deep neural network model to forecast the future trends and prospects of carbon sequestration costs in China’s collective forests.

Third, taking a spatial spillover perspective, this article examines the spatial spillover effects of forest management techniques, socioeconomic development, and forest resource endowment on the carbon sequestration costs of collectively-owned forests. It analyzes how different factors impact carbon sequestration costs across provinces.

## Methodology and data sources

### Carbon sequestration costs in collectively-owned forests

The article employs a net present value model based on opportunity costs to calculate the carbon sequestration costs of collective forests. It is important to clarify that the carbon sequestration costs calculated in this study refer to a series of costs generated by the afforestation project under the afforestation cost method. Drawing from Benítez’s computational approach, the fundamental idea is that the profit from managing forest products should be at least equal to the profit from agricultural products under the same land conditions. Otherwise, the rational decision for operators would be to produce agricultural products rather than forest products. Under conditions where the two profits are equal, the equilibrium state of carbon sequestration prices is determined. This price represents the minimum theoretical cost of conducting forest carbon sequestration operations[22].

Assuming ${F}_{i}$ represents the profit from managing forest products and ${A}_{i}$ represents the opportunity cost, i.e., the profit from agricultural operations, then

1 $${F}_{i}\ge {A}_{i}$$

The profit from managing forest products consists of three components: afforestation costs, timber income, and carbon sequestration income.

2 $${f}_{i}=-{c}_{i}^{a}+\frac{{p}_{i}^{w}{v}_{i}}{{\left(1+{r}_{i}\right)}^{{U}_{i}}}+{B}_{i}$$

In Eq. (2), ${f}_{i}$ represents the net present value of forest management income for plot $i$. ${c}_{i}^{a}$ denotes afforestation costs, ${p}_{i}^{w}$ represents the average market price for timber, ${v}_{i}$ represents timber volume per unit area estimated based on the proportion of collective forest stock, ${r}_{i}$ represents the discount rate or the general social capital return rate, ${U}_{i}$ represents the rotation period of the forest land, and ${B}_{i}$ represents carbon sequestration income.

Taking into account the periodic nature of timber harvesting, the carbon sequestration net present value is calculated based on the rotation period. The carbon sequestration net present value is a function of the present value of carbon sequestration income, the present value of carbon sequestration from forest products, and the carbon emissions during the harvesting period.

3 $${B}_{i}={p}_{i}^{c}\sum_{t=1}^{{U}_{i}}\frac{{\omega }_{i}}{{\left(1+{r}_{i}\right)}^{t}}-\frac{{(1-{\theta }_{i})p}_{i}^{c}{\omega }_{i}{U}_{i}}{{\left(1+{r}_{i}\right)}^{{U}_{i}}}$$

In Eq. (3), ${B}_{i}$ represents the net present value of carbon sequestration from collective forest management during the rotation period. ${\theta }_{i}$ is the carbon sequestration proportion of forest products. ${p}_{i}^{c}$ is the carbon sequestration price. ${\omega }_{i}$ is the yeariy rate of carbon uptake(linear forest growth is considered), calculated based on the conversion equation of forest volume-biomass-NPP (Net Primary Productivity) using data from the National Forest Inventory. Other parameters remain the same as mentioned above. In order to reasonably estimate the carbon sequestration of collective forests, this study incorporates relevant data of collective forests from the National Forest Inventory and applies them to the conversion equation. The conversion equation is as follows:

4 $${\omega }_{i}=\frac{a{V}^{b}\left(1+k\right)S}{cY+d(a{V}^{b}\left(1+k\right)S)}\times 1.63\times \frac{12}{44}$$

Equation (4) represents the volume-biomass-NPP conversion equation, where $V$ denotes the forest volume per unit area of collective forests. $k$ is the root-stem ratio. $Y$ represents the forest age, while $c,d$ are parameters specific to different tree species. $S$ represents the forest area.The coefficient 1.63 is used to convert NPP to CO2, and the coefficient 12/44 is used to convert CO2 to carbon. The selection of these parameters is based on the biomass equation method in the “Forest Carbon Sequestration Management Methodology” and the studies by Liu et al. (2017). The specific values can be found in Table 1. The dominant tree species data for each province are sourced from the “National Forest Inventory.” Moreover, we adopt 50% of NPP as the carbon uptake rate [22].

**Table 1:** Description and Source of Parameters Related to Model

Parameter	Unit	Parameter description and source
${cp}_{i}$	10^4^CNY/hm^2^	Forestry investment/afforestation area, from “the "China Forestry Statistical Yea”book’’
$p{w}_{i}$	CNY/m^3^	Average market price for timber.According to “the ‘‘China Forestry Statistical Yea”book’’
${v}_{i}$	m^3^/hm^2^	Timber volume per unit area of collective forests.According to the National Forest Resources Inventory
${r}_{i}$	%	Bank deposit interest rate for the same period announced by the Monetary Department of the P’ople's Bank of China
${R}_{i}$	Year	Rotation period of the forest land.Tropical forest, subtropical forest, and temperate forest are respectively 20 years, 25 years, and 30 years
$V$	m^3^/hm^2^	Forest volume per unit area of collective forests.According to the National Forest Resources Inventory
$k$	$\%$	Root-stem ratio.Greenhouse Gas Inventory of Land Use Change and Forestry from’hina’s Second National Information Notification
$Y$	Year	Forest age.Take the median age group of different forest types according to “the ‘‘Age Class and Age Group Classification of Main Tree Sp”cies’’
$c$	Constant	Referring to Liu JQ et al. relevant research achievements [44]
$d$	Constant	Referring to Liu JQ et al. relevant research achievements [44]
${\theta }_{i}$	$\%$	Carbon sequestration proportion of forest products.Referring to Zhong WZ et al. research, use the default value of 82.43% [44]
${e}_{i}$	$\%$	Deduction ratio of baseline carbon sequestration.Referring to Benítez et al. research, use the default value of 5%
${A}_{i}$	10^4^ CNY	The ratio of agricultural output value to cultivated land area, from the China Statistical Yearbook

Forest carbon sequestration management must adhere to the principle of additionality, which means the carbon captured above what would have happened. When calculating additional carbon, the baseline carbon needs to be subtracted from the project carbon before calculating the cost of carbon sequestration management. Here, ${e}_{i}$ represents the deduction ratio of baseline carbon sequestration.

Therefore, the final net present value of carbon sequestration management income is given by the following equation:

5 $${B}_{i}={p}_{i}^{c}\left(1-{e}_{i}\right)\sum_{t=1}^{{U}_{i}}\frac{{\omega }_{i}}{{\left(1+{r}_{i}\right)}^{t}}-\frac{(1-{\theta }_{i})(1-{e}_{i}){p}_{i}^{c}{\omega }_{i}{U}_{i}}{{\left(1+{r}_{i}\right)}^{{U}_{i}}}$$

To calculate the revenue for an infinite rotation period based on the given market carbon trading price and rotation cycle, we can use Eqs. (2) and (5).

6 $${F}_{i}={f}_{i}{[{1-(1+{r}_{i})}^{-{U}_{i}}]}^{-1}$$

The opportunity cost of engaging in agricultural production under the same land conditions is determined by the Cobb–Douglas production function in Eq. (7). $K$ and $L$ represent the production factors, while $\alpha$ and $\beta$ denote the output elasticities of the respective factors. $tech$ represents technological progress in production.

7 $${A}_{i}=tech{K}^{\alpha }{L}^{\beta }$$

Substituting Eqs. (5), (6), and (7) into Eq. (1), we can derive the carbon sequestration price for forest carbon management. This price represents the equilibrium cost of carbon sequestration that forest managers aim to achieve equal returns to agricultural production.

8 $$p{c}_{i}=\frac{{A}_{i}\left[1-{(1+{r}_{i})}^{{-U}_{i}}\right]+{c}_{i}^{a}-{p}_{i}^{w}{v}_{i}{(1+{r}_{i})}^{{-U}_{i}}}{{\omega }_{i}\left(1-{e}_{i}\right)\left\{{r}_{i}^{-1}\left[1-{(1+{r}_{i})}^{{-U}_{i}}\right]-{R}_{i}(1-{\theta }_{i}){(1+{r}_{i})}^{{-U}_{i}}\right\}}$$

### Kernel density estimation

Kernel Density Estimation (KDE) is a non-parametric method for estimating probability distributions. It does not rely on prior knowledge and instead fits the distribution based on the data characteristics. The KDE formula is as follows:

9 $$f\left(x\right)=\frac{1}{Nh}\sum_{i=1}^{N}K(\frac{{X}_{i}-x}{h})$$

$N$ represents the number of observations, ${X}_{i}$ denotes independent and identically distributed observations, and $x$ represents the mean value. $Ke$ is the kernel function, and $h$ is the bandwidth. The Gaussian distribution is chosen as the kernel function in this study, which is formulated as:

10 $$Ke\left(x\right)=\frac{1}{\sqrt{2\pi }}{\text{exp}}\left(-\frac{1}{2}{x}^{2}\right)$$

### Deep neural network

**Fig. 1:**
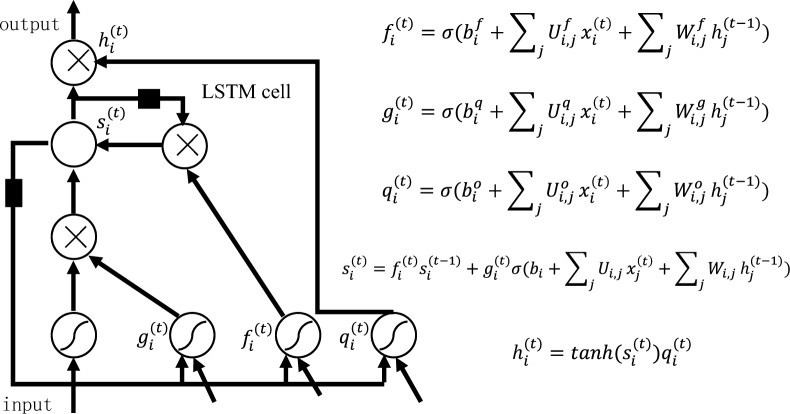
LSTM neural network structure diagram and equation system

We have employed a novel approach utilizing Long Short-Term Memory (LSTM) networks for modeling carbon dynamics within forest ecosystems. LSTM networks offer unique advantages in capturing complex temporal dependencies and nonlinear relationships in the data, facilitating a more accurate understanding of carbon dynamics.The structure of an LSTM network is illustrated in the figure below:

Figure 1 presents the structure and mathematical equations of the LSTM network. The forget gate, denoted as ${f}_{i}^{(t)}$, controls content retention or forgetting from the previous cell. The input gate, represented by ${g}_{i}^{(t)}$, determines which parts of the new cell content are written into the cell. The output gate, ${q}_{i}^{(t)}$, controls the output of cell contents to the hidden state. The hidden state, ${h}_{i}^{(t)}$, reads or outputs content from the final cell. $W$ and $U$ are weight matrices, and $b$ is the bias variable. $h$ is the hidden state. The model has 150 iterations, 50 neurons, and a 5% forget rate. The training data consists of a 2:1 split, with 1992–2011 as the training set and 2012–2021 as the testing set. The trained LSTM model is evaluated on the testing set. Finally, the LSTM model is used to predict the trend of collective forest carbon sequestration costs in China until the carbon peak in 2030.

### Spatial econometric models

Forest carbon sinks exhibit spatial spillover effects due to the interaction of natural endowments, forestry resources, and policies across regions. This means that carbon resources in one province may be spatially correlated with neighboring provinces, impacting the costs of carbon sequestration in collective forests. Given the spatial interdependence in the study domain, the spatial Durbin model is adept at capturing spatial dependence and autocorrelation, thereby enhancing predictive and explanatory capabilities. Furthermore, compared to traditional regression models, the spatial Durbin model better addresses endogeneity and omitted variable issues in spatial data, thereby improving model fit and prediction accuracy.The specific econometric model is as follows:

$$lncc=\mathrm{\alpha }+\uprho Wlncc+{\upbeta }_{0}WTech+{\upbeta }_{1}WSocial+{\beta }_{2}WNature+{\beta }_{4}X+\gamma +\upnu +\upmu$$

11 $$\upmu =\mathrm{\varphi }W\upmu +\upvarepsilon ,\upvarepsilon \sim N\left(0,{\upsigma }^{2}I\right)$$

Equation (10) presents a generalized spatial nested model, known as the spatial Durbin model (SDM). It incorporates the natural logarithm of the collective forest carbon cost (lncc) for each province. The model includes a spatial weight matrix ($W$) and factors such as forestry management technology ($Tech$), socio-economic indicators ($Social$), and natural endowment factors ($Nature$) specific to each province. The set of explanatory variables is represented by $X$.

The model accounts for various effects, including time fixed effects ($\gamma$), individual fixed effects ($\nu$), and a spatial error term ($\mu$). The spatial autocorrelation coefficients are denoted by $\rho$, $\beta$, and $\varphi$, representing the spatial dependency of the dependent variable, independent variables, and error term, respectively.

By constraining the parameters, different spatial dependency conditions can be explored within the spatial econometric framework. For instance, setting $\rho =0$, $\beta =0$, and $\varphi =0$ yields a linear regression model. When $\beta =0$ and $\varphi =0$, the model simplifies to a spatial autoregressive model (SAR). Similarly, if $\rho =0$ and $\beta =0$, it becomes a spatial error model (SEM).

### Data sources

#### Parameters for calculating carbon sequestration costs

The core variable of interest in this study is the carbon sequestration cost of collective forests. During the calculation process, variables accounting for inflation effects were adjusted using a price index with 1992 as the base year. Due to data limitations, the sample excludes Tibet, Hong Kong, Macau, and Taiwan regions. Additionally, the data for Chongqing Municipality is combined with that of Sichuan Province. Therefore, the study focuses on the remaining 29 provinces, covering the period from 1992 to 2021. Missing data for specific years were supplemented using interpolation techniques. The parameters used in the carbon sequestration cost calculation, along with their default values, are presented in Table 1.

#### Factors influencing carbon sequestration costs

Based on existing literature, this study focuses on the core variable of carbon sequestration cost in collective forests. Factors influencing this cost are selected from three aspects: forestry management technology, social economy, and Ecological variables.

Forestry management technology variables: Forestry GDP, timber production, and afforestation area serve as proxies for forestry management technology. Forestry GDP reflects operational effectiveness and development levels in the forestry sector. Timber production indicates regional output levels, with advanced forestry management techniques maintaining optimal forest structure and enhancing growth and yield. Afforestation area reflects expansion and measures production technology. Data for these variables are sourced from the annual ‘‘China Forestry Statistical Yearbook.’’

Socio-economy variables: Labor cost, land use opportunity cost, and rural resident consumption are chosen as proxies for the social economy. Labor cost directly affects carbon sequestration management expenses as it increases. Land use opportunity cost co-varies with carbon sequestration cost due to land scarcity, guiding optimal land allocation. Rural resident consumption provides a comprehensive measure of social and economic factors. Labor cost data are represented by the average salary of forestry employees, while land use opportunity cost is derived from agricultural income under similar conditions. Data for rural resident consumption and land use opportunity cost are obtained from the annual ‘‘China Statistical Yearbook,’’ while labor cost data are sourced from the annual ‘‘China Forestry Statistical Yearbook.’’

Ecological variables variables: Forest stock volume and population density act as proxies for natural environmental factors. Regions abundant in forest carbon sequestration resources are typically remote, with underdeveloped infrastructure and low population density. This may increase the cost of resource development, but also reduce the opportunity cost of labor engagement in forestry due to limited non-agricultural employment opportunities. Forest stock volume data come from the "National Forest Resources Inventory" for the years 1992–2021, with missing data interpolated using natural growth rates. Population density data are obtained from the "China Statistical Yearbook."

The meaning and descriptive statistics of the above variables are shown in Table 2.

**Table 2:** Data description and descriptive statistics

Variable	Unit	Obs	Mean	Std
Carbon sequestration cost	CNY/tco^2^e	870	32.382	35.381
Forestry GDP	billion	870	84.612	97.493
Wood production	10^4^tons	870	251.021	322.912
Afforestation area	hm	870	197.821	175.024
Labor price	CNY	870	4000	3700
Land use opportunity costs	CNY	870	26,000	24,000
Rural residents’ consumption	CNY	870	6476	6843
Forest stock	10^4^m^3^	870	20,000	24,000
Population density	people/km^2^	870	421.011	593.521

#### Overview of collective forests in China

**Fig. 2:**
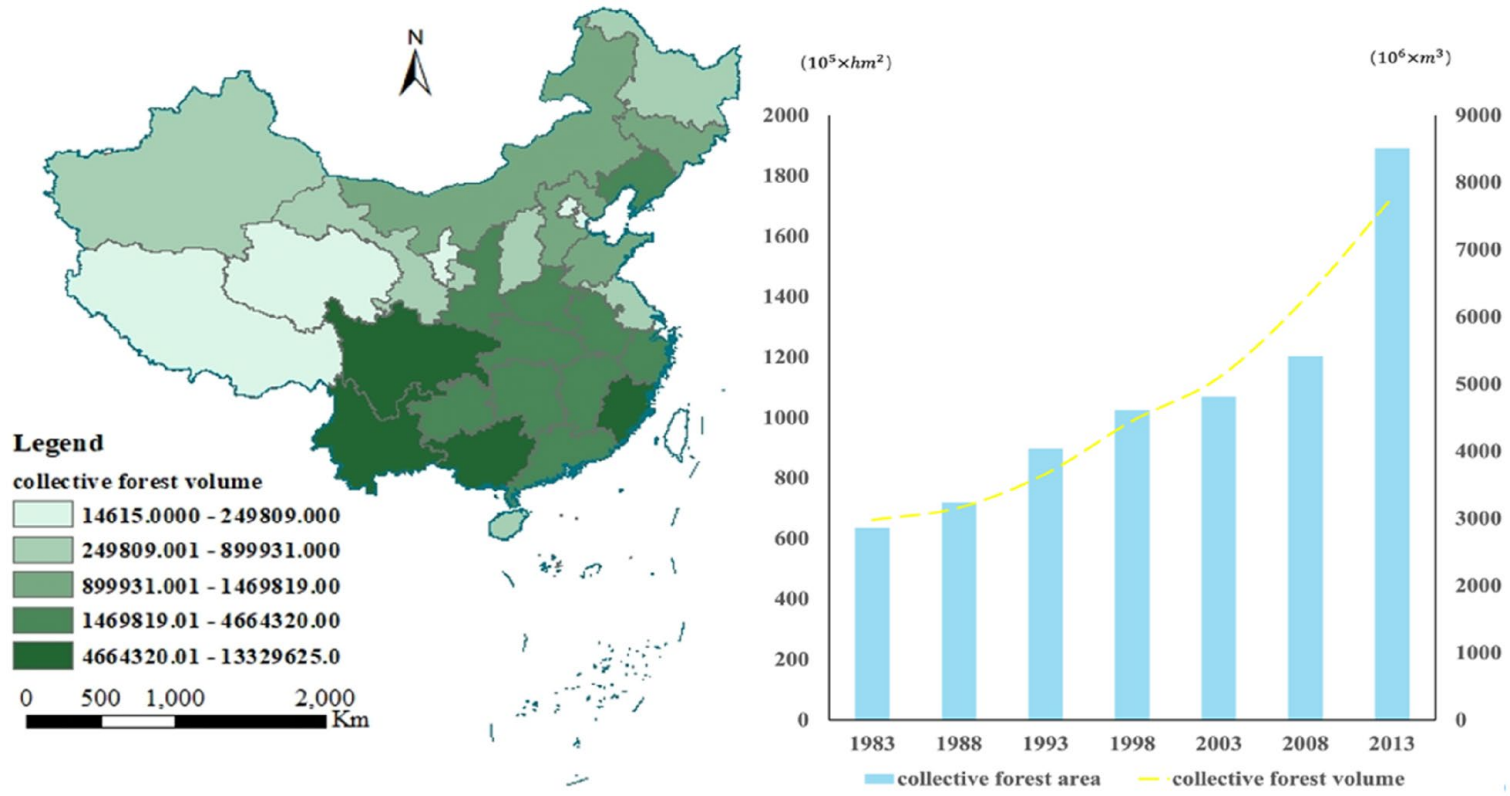
Spatial Distribution and Temporal Changes of Collective Forest Resources in China (Left: Spatial Distribution of Collective Forests from the Ninth National Forest Resources Inventory (2018); Right: Temporal Changes in Area and Volume of Collective Forests in China)

From a spatial perspective, significant disparities exist in the volume of collective forest reserves among different regions (Fig. 2 left). Provinces like Yunnan, Sichuan, Guangxi, and Fujian exhibit higher volumes of collective forest reserves, likely due to their geographical location, climatic conditions, and abundant forest resources. These regions typically boast extensive forest cover, which fosters forest growth and accumulation. Following closely are provinces such as Anhui, Jiangxi, Hubei, Hunan, Gansu, and Zhejiang, where forest reserves range from millions to tens of millions. These provinces form the backbone of collective forest resources in the southern forest region.

Regarding temporal changes, from 1988 to 2018, both the area and volume of collective forest lands have seen continuous growth, with the volume growth rate even surpassing that of the area(Fig. 2 right). This trend likely reflects the growth and accumulation of trees. Particularly notable is the significant acceleration in the growth rate of collective forest lands after 2008, attributed to reforms granting forest management rights to households following the collective forest tenure reform. Overall, these data underscore the effectiveness of measures for protecting and managing collective forest lands and their positive impact on the ecosystem.

## Results

### Dynamic analysis of cost distribution of collective forest carbon sequestration in China

**Fig. 3:**
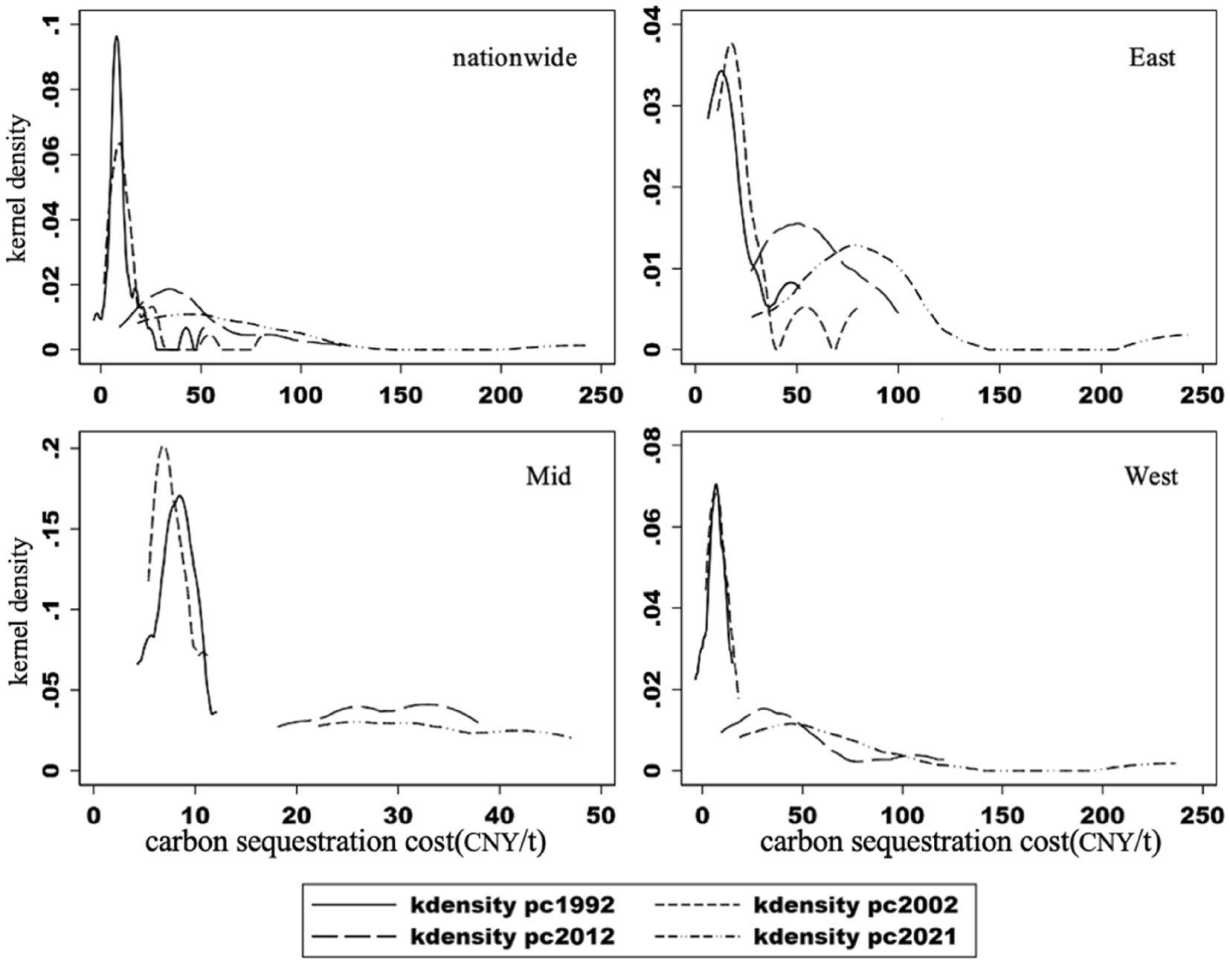
Kernel Density of Carbon Fixation Costs for Collective Forests in Various Regions of China in 1992, 2002, 2012, and 2021

To identify the heterogeneity and dynamic trends of carbon sequestration costs in collective forests at the regional level, this study uses kernel density estimation to plot kernel density maps for different provinces. Figure 3 shows Gaussian kernel density estimates of collective forest carbon sequestration costs in China for 1992, 2002, 2012, and 2021.

Nationally, the peak of the carbon sequestration cost curve gradually decreases, and the range extends to the right. This indicates increasing carbon sequestration costs over time, but with varying magnitudes. The elongated right-tail distribution suggests significant differences among provinces. In 1992 and 2002, the cost curve distributions were concentrated with a single prominent peak, ranging from CNY 0–50 per ton (= 6.42 Euros/t). This indicates relatively low and similar carbon sequestration costs in most provinces' collective forests. In 2012 and 2021, the distributions changed noticeably, with lower peak heights and extended right-tail distributions. The range extended to around CNY 250 per ton (= 32.13 Euros/t), indicating a substantial increase in costs for some provinces, resulting in a wider gap among provinces.

Regionally, the main peak of carbon sequestration cost curves in the eastern region gradually shifted to the right, showing a pattern of “slight increase followed by significant decrease.” The range expanded from CNY 50 per ton in 1992 to nearly CNY 250 per ton in 2021, indicating an overall increase in costs and growing disparity. In the central and western regions, the cost curves were concentrated in 1992 and 2002, with clear peaks ranging from CNY 0–20 per ton (= 0–2.57 Euros/t). In 2012 and 2021, the curves flattened, and in 2021, they shifted further right compared to 2012. However, the central region’s costs remained below CNY 50 per ton, while the western region’s costs extended to CNY 120 per ton (= 15.42 Euros/t) in 2012 and nearly CNY 250 per ton in 2021. This shows a significant increase in costs for some provinces in the western region, leading to internal polarization, while the central region's costs remained relatively low.

### Spatio-temporal evolution of collective forest carbon sequestration costs in China

**Fig. 4:**
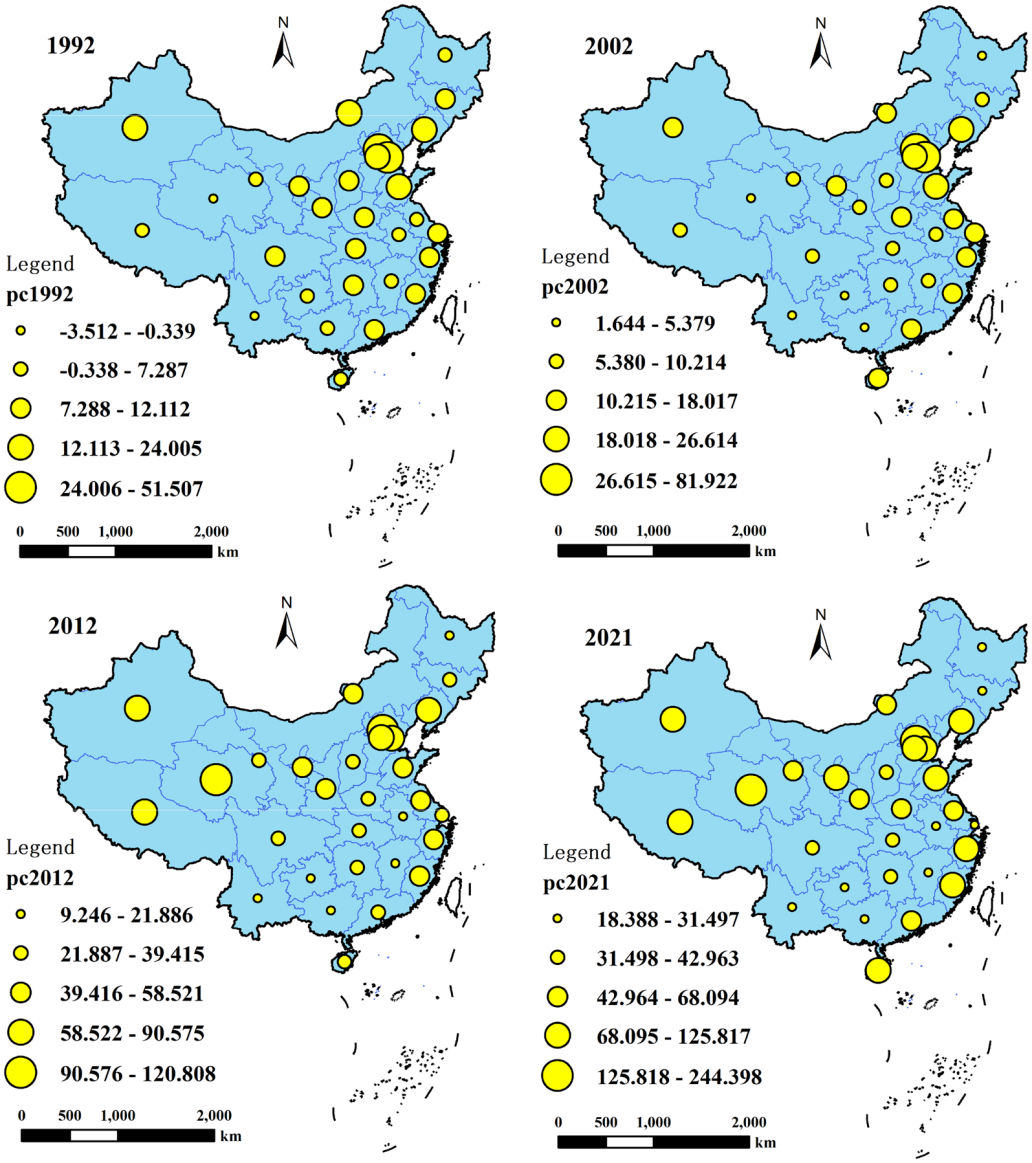
Temporal and Spatial Evolution of Carbon Fixation Costs for Collective Forests in China

To visually illustrate the spatial pattern evolution of collective forest carbon costs, Fig. 4 presents the spatial distribution of collective forest carbon costs in China for the years 1992, 2002, 2012, and 2021. From Fig. 4, it can be observed that the regions with advantageous collective forest carbon costs in China exhibit a west-to-east migration trend.

Regarding the spatial pattern of collective forest carbon costs in 1992, the average national carbon cost is below CNY 50. The Beijing-Tianjin-Hebei region has the highest carbon cost, followed by eastern coastal provinces. The lowest carbon costs are observed in western regions such as Qinghai, Gansu, Yunnan in the southwest, as well as Heilongjiang and Jilin in the northeast. The spatial distribution of carbon costs in 2002 exhibits similar characteristics to 1992, but with an overall increase in carbon costs. Guangxi, Guizhou, and Sichuan provinces experienced relatively decreased carbon costs.

During the late 20th and early twenty-first centuries, China was in a phase of rapid development. A significant labor force migration occurred from west to east, and the eastern regions had already undergone industrialization, leading to increased land and labor prices. This resulted in higher implementation costs for forest carbon in the eastern coastal regions, while carbon costs remained relatively lower in inland provinces.

In 2012, notable changes occurred in the spatial distribution of forest carbon costs. The eastern coastal provinces still had higher carbon costs, while the southern inland, southwest, Heilongjiang, and Jilin provinces experienced a relative decrease in carbon costs. In 2021, the spatial distribution of carbon costs further reinforced these patterns. The southeastern non-coastal regions exhibited a distinct advantage in carbon costs compared to other areas, while the western provinces saw a continuous increase in carbon costs. The west-to-east migration trend of carbon costs between 2012 and 2021 intensified, reversing the distribution pattern of lower costs in the west and higher costs in the east during the 1990s. Currently, the provinces with advantageous collective forest carbon costs are primarily concentrated in the northeastern, central, and southern non-coastal regions.

### LSTM network prediction of China's collective forest carbon sequestration cost

**Fig. 5:**
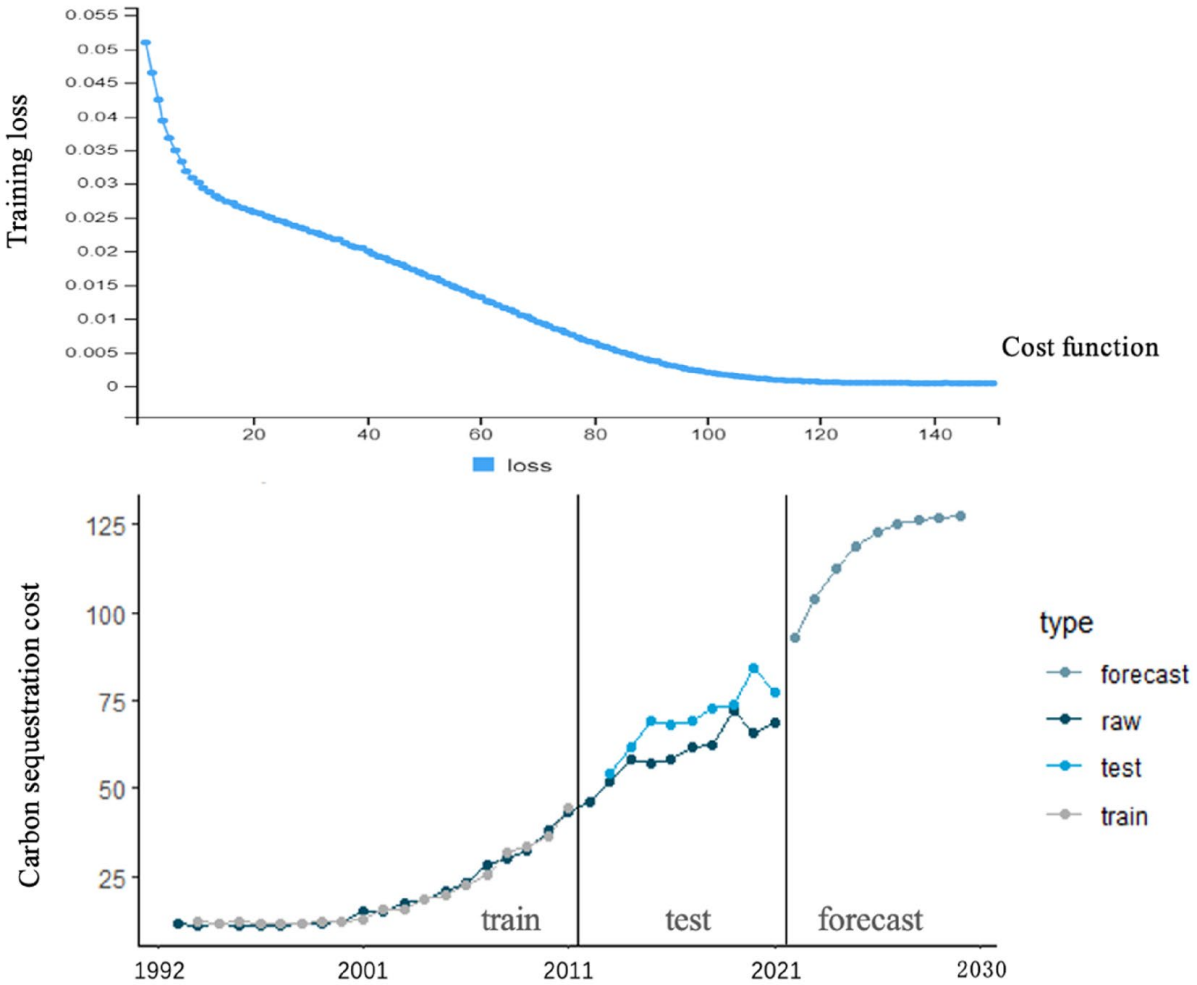
LSTM neural network prediction of the ‘‘carbon peak’’ carbon sequestration cost of collective forests in China in 2023

This study employs the LSTM deep neural network model to forecast the carbon sequestration costs of China's collective forests before the 2030 carbon peak. Figure 5 illustrates the training set, testing set, and future prediction trends of the LSTM model. It demonstrates that the LSTM model accurately predicts the training set, capturing data trends and fluctuations. The testing set predictions also align with the data's changing trends and fluctuations. Overall, the LSTM training results indicate that the network structure learns the data's variations and volatility, enabling predictions of future carbon sequestration cost trends.

The carbon sequestration cost curve exhibits an elongated ‘‘S’’ shape, currently experiencing rapid growth but expected to gradually slow down before the 2030 carbon peak. The projected trend suggests a continuous increase in carbon sequestration costs for collective forests, with an average price of approximately CNY 125 per ton (= 16.06 Euros/t).

The rise in labor force, land use costs, and forest accumulation are significant factors contributing to the continuous growth of carbon sequestration costs for collective forests. As the policy-driven carbon peaking process advances, the demand for forest carbon sinks in the carbon market increases. However, limited available land for afforestation as a carbon sink, due to land resource constraints, necessitates a shift towards intensive management of existing carbon sink forests. This shift results in increased operational costs for forest carbon sequestration. Furthermore, from an alternative perspective, as industrial emission reduction costs increase with emission volume, enterprises seek more cost-effective alternatives. Forest carbon trading, compared to engineering emission reductions, emerges as a preferable option, leading to an increase in forest carbon sequestration costs. Additionally, macroeconomic conditions impact forest carbon sequestration costs. Currently, there is a low return on social capital, and major developed countries have entered a zero interest rate era. Changes in interest rates affect the calculation of net present value for forest carbon sinks, contributing to increased carbon sequestration costs.

### Spatial spillover effects of collective forest carbon sequestration costs in China

#### Benchmark regression results

The regression results of the panel data with a two-way fixed effects model, ignoring spatial correlation, are presented in Table 3. The results indicate significant negative effects of forest accumulation, forestry gross value added, and timber production on the carbon sequestration costs of collective forests. Land opportunity cost, rural household consumption, population density, and afforestation area show significant positive effects on carbon sequestration costs. However, the impact of labor force price on carbon sinks in collective forests is not significant. The results of the spatial correlation test, as indicated by the robust LM test statistics, are all significant at the 1% level, suggesting that the model specifications cannot reject the presence of spatial lag and spatial error effects.

**Table 3:** Regression results of non spatial correlation panel data econometric model of carbon sequestration cost

	(1)	(2)		(3)	(4)
Forestry GDP	− 8.493^***^	− 24.873^***^		− 7.822^***^	− 25.456^***^
	(− 6.935)	(− 8.753)		(− 5.914)	(− 6.817)
Wood production	− 0.081	− 3.702^***^		− 0.626	− 4.568^***^
	(− 0.137)	(− 5.130)		(− 1.010)	(− 6.123)
Afforestation area	9.900^***^	1.429		11.842^***^	1.828^*^
	(12.300)	(1.633)		(12.932)	(1.889)
Labor Price	− 0.309	− 2.558^***^		2.929^*^	− 2.178
	(− 0.294)	(− 2.787)		(1.866)	(− 1.496)
Land use costs	34.337^***^	39.607^***^		37.198^***^	57.210^***^
	(18.260)	(14.441)		(18.004)	(13.606)
Rural residents’ consumption	7.728^***^	16.576^***^		15.485^***^	19.739^***^
	(5.128)	(6.875)		(4.928)	(4.336)
Forest stock	− 7.281^***^	− 12.033^***^		− 7.291^***^	− 11.142^***^
	(− 11.530)	(− 11.610)		(− 11.462)	(− 10.635)
Population density	− 2.961^***^	47.994^***^		− 4.093^***^	55.289^***^
	(− 4.486)	(6.321)		(− 5.281)	(6.404)
Year FE	No	No		Yes	Yes
Prov FE	No	Yes		No	Yes
*N*	870	870		870	870
r^2^	0.668	0.831		0.683	0.844
Spatial error Lagrange multiplier			0.409		
Spatial error Robust Lagrange multiplier			18.284^***^		
Spatial lag Lagrange multiplier			11.973^***^		
Spatial lag Robust Lagrange multiplier			29.848^***^		

The significance level is *p < 0.1, **p < 0.05, ***p < 0.01, with t-statistic in parentheses. Year FE represents a fixed time effect, while Prov FE represents a fixed provincial effect, as shown in the following table

#### Spatial Durbin model regression results

The LM test conducted earlier revealed the presence of spatial effects among sample entities, indicating a potential misspecification issue in the conventional panel data model. Therefore, we employed the spatial Durbin model to analyze the factors influencing carbon sequestration costs in China's collective forests and their spatial spillover effects. The regression results of the spatial Durbin model are presented in Table 4.

**Table 4:** Regression Results of Panel data Spatial Durbin Model of Carbon Fixation Cost

	(1)	(2)	(3)	(4)
Forestry GDP	− 28.446^***^	− 26.356^***^	− 6.734^***^	− 24.152^***^
	(− 9.421)	(− 7.892)	(− 5.000)	(− 6.836)
Wood production	− 2.317^***^	− 3.206^***^	− 0.700	− 5.044^***^
	(− 3.041)	(− 4.487)	(− 1.160)	(− 7.332)
Afforestation area	2.310^**^	1.259	10.483^***^	0.610
	(2.413)	(1.389)	(12.124)	(0.687)
Labor Price	0.947	− 0.749	5.134^***^	− 0.438
	(0.733)	(− 0.616)	(3.507)	(− 0.323)
Land use costs	47.626^***^	46.831^***^	31.860^***^	54.240^***^
	(11.947)	(11.590)	(14.037)	(13.613)
Rural residents' consumption	7.795^*^	11.268^***^	20.160^***^	16.143^***^
	(1.777)	(2.682)	(5.876)	(3.724)
Forest stock	− 10.287^***^	− 10.969^***^	− 6.842^***^	− 11.136^***^
	(− 9.532)	(− 10.568)	(− 10.958)	(− 11.258)
Population density	16.831^**^	47.243^***^	− 3.590^***^	68.675^***^
	(2.156)	(5.693)	(− 4.879)	(8.156)
W × Forestry GDP	4.256	0.002	14.980^***^	0.220
	(0.900)	(0.000)	(5.423)	(0.027)
W × Wood production	− 5.278^***^	− 4.414^***^	− 7.638^***^	− 11.437^***^
	(− 4.869)	(− 4.059)	(− 7.687)	(− 9.322)
W × Afforestation area	− 1.075	− 0.412	5.172^***^	− 0.992
	(-0.780)	(− 0.303)	(2.687)	(− 0.569)
W × Labor Price	− 2.858^**^	− 1.727	-0.901	− 5.460^**^
	(− 1.992)	(− 1.244)	(− 0.321)	(− 2.163)
W × Land use costs	− 3.391	− 1.505	− 1.748	53.097^***^
	(− 0.599)	(− 0.262)	(− 0.356)	(5.830)
W × Rural residents’ consumption	8.589	6.449	39.658^***^	24.594^***^
	(1.630)	(1.233)	(5.497)	(2.696)
W × Forest stock	4.829^**^	4.244^**^	4.522^***^	6.333^***^
	(2.452)	(2.098)	(3.052)	(2.923)
W × Population density	− 14.398	− 48.095^***^	− 9.381^***^	11.131
	(− 1.472)	(− 3.008)	(− 6.696)	(0.579)
Spatial rho	− 0.077	− 0.049	− 0.132^**^	− 0.220^***^
	(− 1.272)	(− 0.828)	(− 2.148)	(− 3.576)
Year FE	No	No	Yes	Yes
Prov FE	No	Yes	No	Yes
*N*	870	870	870	870
r^2^	0.379	0.214	0.522	0.240

Table 4 shows that the spatial autoregressive coefficient for carbon sequestration costs is significant, indicating notable spatial correlation among neighboring provinces. Forest accumulation, forestry gross value added, and timber production have significant negative effects on carbon sequestration costs in the respective provinces. On the other hand, land opportunity cost, rural household consumption level, and population density exhibit significant positive effects on carbon sequestration costs.

Provinces with higher forest accumulation, representing abundant forest resources, experience lower carbon sequestration costs, demonstrating a "diminishing marginal cost" nature of carbon sequestration benefits. Provinces with higher forestry gross value added and timber production tend to have advanced forestry techniques and operational efficiency, leading to lower carbon sequestration costs.

Land, as a necessary input for carbon sequestration in collective forests, becomes more constrained and costly when its opportunity cost rises due to alternative economic activities such as industrial or urban development. The increased rural household consumption and population density indicate resource competition and limited land supply, resulting in higher competition costs for collective forest carbon sequestration.

The spatial lagged terms of the independent variables, including forest accumulation, land opportunity cost, timber production, rural household consumption, and labor force price, exhibit significant spatial spillover effects. Notably, the indirect spatial spillover effect of forest accumulation differs from the direct effect discussed earlier, while the indirect effect of labor force price is significant despite the insignificant direct effect.

Additionally, we examined the influence of unobservable factors on the model results. Model (4), which includes two-way fixed effects, shows larger absolute values for regression coefficients compared to Model (2), which only considers individual fixed effects. This suggests the presence of policy shocks or time-varying factors that have led to an underestimation of the spatial spillover effects on carbon sequestration costs. Moreover, compared to Model (3) with only time fixed effects, Model (4) incorporating spatial lagged terms demonstrates increased absolute coefficients, highlighting the importance of accounting for individual differences to avoid underestimating the spatial spillover effects on carbon sequestration costs.

#### Spatial spillover decomposition

The spatial econometric model analyzed in the previous section indicates that the coefficient of the spatial lag term does not directly reflect the marginal changes of the independent variables on the dependent variable. To explain the marginal effects of the independent variables from neighboring provinces on the dependent variable, namely spatial spillover effects, it is necessary to decompose the coefficients into direct and indirect effects. Table 5 presents the results of the spatial spillover effects decomposition.

**Table 5:** Decomposition of direct and indirect effects of carbon sequestration cost spatial spillover

	Total effect		Indirect effect		Direct effect	
	Effect	T-value	Effect	T-value	Effect	T-value
Forestry GDP	− 19.423^**^	− 2.377	4.205	0.646	− 23.628^***^	− 6.848
Wood production	− 13.537^***^	− 11.726	− 8.252^***^	− 8.228	− 5.285^***^	− 7.870
Afforestation area	− 0.365	− 0.227	− 0.939	− 0.688	0.574	0.623
Labor Price	− 4.871^**^	− 2.009	− 4.271^**^	− 2.132	− 0.600	− 0.471
Land use costs	88.601^***^	11.063	33.567^***^	4.647	55.034^***^	14.363
Rural residents’ consumption	33.242^***^	3.817	16.724^**^	2.244	16.519^***^	4.049
Forest stock	− 3.775^**^	− 2.051	7.122^***^	4.335	− 10.896^***^	− 10.794
Population density	65.900^***^	3.706	− 3.527	− 0.219	69.427^***^	8.254

The decomposition of indirect effects reveals the following: Firstly, both the timber production and labor price exhibit negative spatial spillover effects, indicating that an increase in timber production and labor price in neighboring provinces would reduce the carbon sequestration cost of collective forests in the focal province. An increase in timber production in neighboring provinces may lead to a higher inflow of timber into the focal province, thereby restraining its timber harvesting. Given the established forest age structure and forest management inputs, a lower timber harvesting volume results in higher carbon sequestration in forests, leading to a reduction in carbon sequestration costs of collective forests in the focal province [36]. Labor is mobile, and an increase in labor prices in neighboring provinces creates a suction effect on labor outflow from the focal province, which contributes to the scale effect of forest management through land circulation. Consequently, it leads to a decrease in carbon sequestration costs of collective forests.

Secondly, the opportunity cost of land use, rural household consumption, and forest stock exhibit positive spatial spillover effects on the carbon sequestration costs of collective forests. The rise in land use opportunity cost is often associated with urbanization. If a particular region's land use shows higher economic benefits, surrounding areas may also be influenced as resources are attracted to that location, thereby increasing the opportunity cost of land use and, consequently, the cost of carbon sequestration.Forests not only provide timber as building materials but also offer ecological products, such as protecting water sources, purifying air, and maintaining biodiversity. Areas in the later stage of urbanization have a higher demand for ecological products and pay more attention to the environment, thereby emphasizing the protection of forest resources. Consequently, it leads to higher carbon sequestration costs [15]. The upgrading of rural household consumption in neighboring provinces is usually accompanied by an increase in demand for ecological and timber products, triggering resource competition. Competition, in turn, raises the prices of factors of production for collective forest management, resulting in an increase in carbon sequestration costs in the focal province[43]. The forest stock in a specific province partly reflects the effectiveness of forest conservation policies and quota-based harvesting systems. A higher forest stock in neighboring provinces may be the result of forestry policies, which induces an increase in forest harvesting in the focal province and consequently leads to an increase in carbon sequestration costs [45].

### Robustness check

The benchmark regression and spatial Durbin model regression results in this study both indicate the presence of spatial spillover effects in the carbon sequestration costs of collective forests. However, there may still be some underlying factors that could affect the robustness of the regression results. Therefore, this study conducts robustness tests on the specification of the spatial weight matrix in the spatial econometric model by constructing geographic distance and economic distance matrices. The aim is to examine and decompose the spatial spillover effects of the spatial Durbin model.

After replacing the spatial weight matrix with the economic distance and geographic distance matrices, the spatial Durbin model is once again used to decompose the spatial spillover effects. Other model specifications remain consistent with the previous analysis. The results, as presented in Table 6, demonstrate that the spatial spillover effects decomposition results of the spatial Durbin model under the specifications of the economic distance matrix and geographic distance matrix are consistent with the previous findings obtained using the adjacency spatial matrix.

**Table 6:** Robustness test results of other spatial weight matrice

	Economic distance matrix			Geographic distance matrix		
	Direct	Indirect	Total	Direct	Indirect	Total
Forestry GDP	− 21.524^***^	− 0.879	− 22.403^***^	− 21.524^***^	− 0.879	− 22.403^***^
Wood production	− 2.355^***^	3.828^***^	1.473	− 2.355^***^	3.828^***^	1.473
Afforestation area	4.314^***^	− 4.445^***^	− 0.132	4.314^***^	− 4.445^***^	− 0.132
Labor Price	− 2.468^**^	2.565	0.097	− 2.468^**^	2.565	0.097
Land use costs	55.929^***^	20.436^***^	76.365^***^	55.929^***^	20.436^***^	76.365^***^
Rural consumption	19.591^***^	− 37.330^***^	− 17.739^***^	19.591^***^	− 37.330^***^	− 17.739^***^
Forest stock	− 8.163^***^	7.800^***^	-0.364	− 8.163^***^	7.800^***^	− 0.364
Population density	59.884^***^	− 53.651^***^	6.233	59.884^***^	− 53.651^***^	6.233

The significance level is ^*^ p<0.1, ^* *^ p<0.05, ^* * *^ p<0.01, with t-statistic in parentheses

## Conclusion

This study estimates the changes in carbon sequestration costs of collective forests in China over the past 30 years, using the forest carbon sequestration cost model. It analyzes the spatiotemporal evolution characteristics and predicts the carbon sequestration costs of collective forests before the carbon emissions peak in 2030 using a deep neural network model. The spatial Durbin model is applied to explore the spatial spillover effects of carbon sequestration costs. The key research findings are as follows:

The regions with advantages in carbon sequestration costs of collective forests in China have shifted from west to east over the past 30 years. Initially, cost advantages were mainly concentrated in the northwest and northeast provinces, while in the later period, the southern collective forest areas, excluding coastal provinces, had lower carbon sequestration costs.

The carbon sequestration costs of collective forests in China have shown an increasing trend since 1992. Predictions indicate that by 2030, the average carbon sequestration cost of collective forests in China will reach CNY 125 per ton(= 16.06 Euros/t).

Forest stock volume, total forestry production value, and timber production have a significant negative impact on the carbon sequestration costs of collective forests in respective provinces. Conversely, land opportunity costs, rural resident consumption levels, and population density have a significant positive impact on the carbon sequestration costs.

Carbon sequestration costs exhibit significant spatial correlation. Land opportunity costs, rural resident consumption levels, and forest stock volume have positive spatial spillover effects on the carbon sequestration costs of collective forests. Conversely, timber production and labor costs have significant negative spatial spillover effects.

Based on the research findings, the following policy implications are derived:

Priority should be given to developing forest carbon sink resources in southern collective forest areas, which currently have lower carbon sequestration costs. This involves encouraging carbon sequestration projects, promoting afforestation on suitable land, incentivizing carbon sequestration management on existing forested land, and enhancing forest carbon absorption.

Efforts are needed to balance the utilization and protection of forest resources and to strengthen the sustainable management of collective forests. This includes adopting sustainable forest management measures to improve timber production efficiency and quality, reduce waste and losses, and decrease carbon sequestration costs. Additionally, forest resource management and protection should be enhanced, encompassing improved forest management planning, conservation, and restoration projects, as well as sustainable forestry operations aimed at increasing forest stock volume.

Interprovincial forestry exchange should be enhanced to coordinate regional forest resource development and afforestation activities. Given the spatial spillover effects of carbon sequestration costs in collective forests, promoting cross-regional cooperation and coordination is essential to facilitate resource sharing and flow. It is crucial to foster resource sharing and technology transfer to reduce carbon sequestration costs and achieve synergistic effects between regions, ultimately improving overall carbon sequestration benefits.

In methodology, Long Short-Term Memory (LSTM) networks outperform traditional forecasting models by capturing nonlinear factors in the carbon market. This enhances our ability to predict carbon sequestration costs, considering the market's complexity and uncertainty comprehensively. By adeptly handling various nonlinear relationships, we aim for a more accurate portrayal of underlying patterns and regularities within the carbon market, providing carbon market participants with effective decision support and risk management strategies.

## Data Availability

The data sources have been indicated in the text, and all data are available upon request from the authors.
